# Sex Differences in Mental Status and Coping Strategies among Adult Mexican Population: A Cross-Sectional Study

**DOI:** 10.3390/healthcare11040514

**Published:** 2023-02-09

**Authors:** Aniel Jessica Leticia Brambila-Tapia, Fabiola Macías-Espinoza, Joel Omar González-Cantero, Reyna Jazmín Martínez-Arriaga, Yesica Arlae Reyes-Domínguez, María Luisa Ramírez-García

**Affiliations:** 1 Departamento de Psicología Básica, Centro Universitario de Ciencias de la Salud (CUCS), Universidad de Guadalajara, Guadalajara 44340, Jalisco, Mexico; 2 Departamento de Psicología Aplicada, Centro Universitario de Ciencias de la Salud (CUCS), Universidad de Guadalajara, Guadalajara 44340, Jalisco, Mexico; 3 Departamento de Ciencias del Comportamiento, Centro Universitario de los Valles (CUVALLES), Universidad de Guadalajara, Guadalajara 46600, Jalisco, Mexico; 4 Clínicas de Salud Mental, Centro Universitario de Ciencias de la Salud (CUCS), Universidad de Guadalajara, Guadalajara 44340, Jalisco, Mexico; 5 Maestría en Psicología de la Salud, Centro Universitario de Ciencias de la Salud (CUCS), Universidad de Guadalajara, Guadalajara 44340, Jalisco, Mexico

**Keywords:** stress, coping strategies, anxiety, depression, sex

## Abstract

We performed a cross-sectional study in order to determine the association between stress coping strategies and stress, depression, and anxiety, in which the Mexican population was invited to answer these variables by an electronic questionnaire. A total of 1283 people were included, of which 64.8% were women. Women presented higher levels of stress, depression, and anxiety than men; likewise, women showed a higher frequency of some maladaptive coping strategies (behavioral disengagement and denial) and lower levels of some adaptive ones (active coping and planning); additionally, maladaptive coping strategies were positively correlated with stress and depression in both sexes: self-blame, behavioral disengagement, denial, substance use, and self-distraction. Likewise, there were negative correlations between stress and depression and the adaptive strategies: planning, active coping, acceptance, and positive reframing. For women, religion presented negative correlations with stress, depression, and anxiety, and humor showed low positive correlations with stress, anxiety, and depression. In conclusion, most adaptive and maladaptive coping strategies are common in both sexes with the exception of religion, which seems to be adaptive in women and neutral in men, and humor, which seems to be adaptive in men and maladaptive in women. In addition, emotional and instrumental support seem to be neutral in both sexes.

## 1. Introduction

Coping strategies are defined as an individual’s attempts to use cognitive and behavioral strategies to manage and regulate pressures, demands, and emotions in response to stress [1]; which, in turn, is considered a “particular relationship between the person and the environment that is appraised by the person as taxing or exceeding his or her resources and endangering his or her well-being” [2].

Stress has been associated with a wide number of negative psychological variables including depression, anxiety, and somatization; for example, a positive moderate correlation between stress and somatization has been reported [3,4,5]. In addition, many reports have shown that all these negative psychological variables have been observed at higher levels in women than in men [4,5,6]. These differences have been explained by the presence of progesterone during the luteal phase in fertile women [7], and by the dysregulation of glucocorticoid receptors, which was observed in female rats [8]; however, it is possible that more molecular, cultural, and psychological explanations may be involved. 

Additionally, stress has been associated with elevated levels of blood pressure, serum lipids, reactive C protein, and oxidative stress [9,10,11,12], which is why it has been associated with the appearance of chronic diseases.

Furthermore, it has been reported that men and women show different associations between some coping strategies and psychological and physiological variables [13,14,15,16]. In this sense, Blalock & Joiner [13] showed that cognitive avoidance, as a coping style, was associated with higher depression and anxiety in response to stressful life events in women when compared with men; in addition, this coping strategy was associated with lower levels of systolic blood pressure only in men [14]. Likewise, Kelly et al. [16] showed that self-blame was associated with more anxiety-trait in women and that positive restructuring was associated with less depression also in women in comparison with men. Additionally, Mazure & Maciejewski [15] showed that women are sensitive to a broader range of stressful life events than men and that in response to these, women are three times more prone to experience depression than men. This information supports the contention that women show higher responses to stress than men and that specific coping styles are more maladaptive in women than in men. 

With respect to stress coping styles, there are a few instruments that measure them. The main ones are the Coping Strategies Inventory (CSI) [17] and the Brief-COPE [18]; these instruments share common coping strategies, but the Brief-COPE is the instrument that incorporates the measurement of more coping strategies than CSI, i.e., 14 strategies for the Brief-COPE vs. 8 strategies for the CSI. In this regard, Meyer [19] classified the stress coping strategies of the Brief-COPE instrument into two large dimensions: adaptive (including the use of emotional support, positive reframing, acceptance, spirituality/religion, humor, active coping, planning, and use of instrumental support) and maladaptive coping strategies (including denial, behavioral disengagement, self-blame, substance use, self-distraction, and venting). However, Cooper et al. reduced the Brief-Cope instrument to three dimensions: emotional-focused strategies (acceptance, emotional support, humor, positive reframing, and religion), problem-focused strategies (active coping, instrumental support, and planning), and dysfunctional-focused strategies (behavioral disengagement, denial, self-distraction, self-blame, substance use, and venting) [20].

However, none of these two main classifications has been proposed based on correlations with stress or any negative psychological variable; therefore, in this study, we have two main objectives: (1) to compare the different coping strategies by sex, in order to detect any potential difference that could explain the different responses to stress in each sex and their relation with the presence of stress, depression, and anxiety; and (2) to investigate the relationship of each coping strategy (of the Brief-COPE instrument) with the main psychological variables: stress, anxiety, and depression, according to sex, in order to determine the adaptive and maladaptive coping strategies in each sex, corroborated from an empirical approach. 

This study has, therefore, two main hypotheses: (a) women present higher levels of maladaptive coping strategies and lower levels of adaptive strategies than men, and (b) the coping strategies considered as adaptive by Meyer show negative correlations with stress, depression, and anxiety; and those classified as maladaptive or dysfunctional strategies show positive correlations with stress, depression, and anxiety.

The study of these two important objectives, not previously performed, in a sample of the Mexican population would add important and useful information to the international literature on the topic, either for research or practice.

## 2. Subjects and Methods

This is a cross-sectional study performed in the period from September 2021 to March 2022. The target population was any adult (older than 18 years old) who resided in Mexico. The procedure consisted of the distribution of an electronic questionnaire that included sociodemographic variables and psychological instruments, which was sent via social networks (including Facebook, WhatsApp, e-mail, and the classroom application of Google). The reached population included university students, colleagues, and acquaintances of the research team in the first step and a more diverse population in the second step, given that the instrument was widely distributed with the snowball method. The study was conducted in accordance with the declaration of Helsinki and was approved by the ethics and research committee of the Health Sciences University Center (CUCS) of the University of Guadalajara (number: CI-06821). All the participants gave their consent to participate in the same questionnaire.

### 2.1. Sampling Strategy and Sample Size

The sampling approach was the snowball method, which corresponds to a non-random method, and the sample size was expected to be a minimum of 500 participants, in order to detect very low correlations (r < 0.2) as significant and add more potentially useful information to the international literature, considering that very low correlations can be useful in different research and practice contexts.

### 2.2. Variables Included

The socio-demographic data included age, sex, schooling, whether participants have a romantic partner, whether they have a job, and their socioeconomic level. 

The psychological measures included stress, measured with the Cohen Perceived Stress Scale (CPSS) [21,22]; this scale consists of 14 questions with 5 answer options from “never” to “very frequently” (Cronbach´s alpha test: 0.855); depression was measured with the CES-D Scale (Cronbach’s alpha test: 0.867) [23,24], which consists of 10 questions with 4 answer options from “none day” to “5–7 days in the week”; anxiety was measured with the GAD-7 Scale (Cronbach’s alpha test: 0.923) [25,26]; this instrument consists of 7 questions with 4 answer options from ”never” to “almost all the days”. Coping strategies were measured with the Brief-COPE Scale; Cronbach´s alpha tests were above 0.6 for most subscales and above 0.5 for acceptance, self-distraction, behavioral disengagement, and denial. However, the subscale “venting” had a low Cronbach’s alpha of 0.35, and therefore this subscale was not used for the analyses [18,27]. This instrument consisted of 28 questions with 4 answer options from “I never do it” to “I always do it”, and in addition, this instrument presents 14 subscales (2 questions for each subscale).

We used the Spanish adaptations of scales because no Mexican adaptations were found; however, Spanish adaptations were considered understandable and adequate for the Mexican population and showed a desirable internal consistency in previous publications performed by the research group.

### 2.3. Statistical Analysis

In order to describe qualitative or categorical variables, we used frequencies and percentages and to describe quantitative variables we used means, and standard deviation; these were used instead of median and ranges (considering the non-parametric distribution of the data) because they better reflected the differences observed between sexes. In order to verify the distribution of the data, we used the Kolmogorov–Smirnov test. In order to compare categorical variables between sexes we used a chi-squared test. To compare the psychological variation between sexes, we used the Man–Whitney U test, considering the non-parametric distribution of these variables. To perform correlations between psychological variables, we used the Spearman correlation test. Finally, a multiple regression analysis, with the stepwise method for stress as a dependent variable, was performed for each sex, in order to determine the variables significantly correlated with stress after adjustment for confounders in both sexes. In this analysis, we excluded the variables anxiety and depression (in order to detect the coping strategies most associated with stress, excluding the variables most associated with stress: anxiety and depression). All analyses were performed with the software SPSS v. 25, and a *p* value < 0.05 was considered significant.

## 3. Results

A total of 1283 participants were included, which represents an approximate 25.6% response rate (considering that the survey reached around 5000 persons); we eliminated the responses of participants that seemed to be incongruent in the data reported (i.e., those responses that included the same number in many questions, being even contradictory). No missing data were reported because all questions were marked as obligatory. 

Of the included participants, 831 (64.8%) were women, and the sociodemographic data of participants are presented in Table 1. There were no differences in age, schooling, having a romantic partner, having a job, or socioeconomic level between the sexes. 

### 3.1. Comparison of Psychological Variables and Coping Strategies between Sexes

In Table 2 we show the means and standard deviations of coping strategies for each sex. We observed that the most frequent coping strategies in both sexes were acceptance, active coping, and planning while the least frequent were substance use, denial, and behavioral disengagement. 

In the comparison of the psychological variables by sex, we observed that women had significantly higher levels of stress, anxiety, and depression. In relation to the coping strategies, women had higher levels of religion, behavioral disengagement, emotional support, and denial and lower levels of acceptance, humor, planning, and active coping, when compared with men (Table 2). 

### 3.2. Bivariate Correlations

In the correlations of the coping strategies with stress, anxiety, and depression by sex, we observed that, in both sexes, there were positive correlations between stress, depression, and anxiety and the following strategies: self-blame, behavioral disengagement, denial, substance use, and self-distraction. Likewise, in both sexes, there were negative correlations between stress, depression, and anxiety with planning, active coping, acceptance, and positive reframing. For women, religion also presented negative correlations with stress, depression, and anxiety that were not found in men. In addition, in men, a very low but significant negative correlation was found between humor and stress. However, in women, humor presented very low but positive significant correlations with stress, depression, and anxiety. In the case of emotional and instrumental support, they did not show significant correlations with stress or depression in any sex (Table 3).

Some sociodemographic variables also showed low negative but significant correlations with stress in both sexes, which are shown in Table 4.

### 3.3. Multivariate Analysis for Stress

In the multiple regression analysis for stress by sex, and adjusting for sociodemographic variables, we observed that self-blame was the variable most positively related to stress in both sexes. In the case of women, other positively correlated variables were detected: denial, behavioral disengagement, and self-distraction, while in men other positively correlated variables were denial, self-distraction, and substance use. The negatively associated variables with stress in women were active coping, acceptance, and positive reframing, while in men they were active coping, acceptance, and humor. In addition, some sociodemographic variables were also negatively associated with stress, including age and having a job in women and in men: schooling and socioeconomic level (Table 5 and Table 6).

## 4. Discussion

As previously reported, higher levels of stress, depression, and anxiety were found in women when compared with men [3,4,5,6]. A factor possibly related to these differences is the higher prevalence of some maladaptive coping strategies (behavioral disengagement and denial) and the lower frequency of some adaptive ones (acceptance, active coping, and planning) in women than in men; these results corroborate our first hypothesis. However, religion showed a higher frequency in women, in whom it was negatively correlated with stress, depression, and anxiety, and humor was higher in men, in whom it was negatively related to stress, indicating that these two strategies (religion and humor) showed higher frequencies in the sex where they were more adaptive. Nevertheless, it is noteworthy that adaptive strategies were the most frequent coping strategies and maladaptive ones were the least frequent coping strategies in both sexes.

With respect to the analysis of correlations, we observed that in contrast to a previous report, which showed that avoidance coping was not associated with an increase in depression and anxiety in men, and another one showing that avoidance coping was actually associated with lower blood pressure in men [13,14], we found that avoidance coping (denial, self-distraction, and behavioral disengagement) showed positive correlations with stress, depression, and anxiety in both sexes. However, the most associated variable with stress, depression, and anxiety was self-blame. These correlations coincide with our previous report where the coping strategies associated with stress, depression, and anxiety were associated with somatization in both sexes, and some of them were also associated with a number of diseases, with the highest association for self-blame [3]. These results coincide with our second hypothesis, confirming that coping strategies classified as maladaptive by Meyer showed positive correlations with stress, depression, and anxiety, while most classified as adaptive ones showed negative correlations with these three variables. The only difference with respect to Meyer’s classification is related to the coping strategies of religion and humor, which showed different correlations in each sex.

These associations are further corroborated in the multiple regression analysis performed by sex, where common maladaptive coping strategies were significantly associated with stress in both sexes (self-blame, denial, and self-distraction), with self-blame the most associated variable with stress. These results coincide with a meta-analysis performed among healthcare professionals, which showed that maladaptive coping strategies related to poor mental health outcomes were venting, denial, disengagement, self-blame, and substance use; they also found personal factors related to the use of maladaptive coping strategies, including being female, older than 50 years old, living alone, and having a history of personal trauma. In addition, environmental factors related to the use of these strategies were work stress, workload, and poor benefits [28]. This data also shows that environmental conditions are related to the use of adaptive or maladaptive coping strategies.

Based on these results, the adaptive strategies in both sexes would be active coping, planning, acceptance, and positive reframing; in addition, religion is also adaptive in women and humor in men. Maladaptive coping strategies in both sexes would be self-blame, behavioral disengagement, self-distraction, denial, and substance use; in addition, humor would be also maladaptive in women. Finally, neutral strategies would be emotional and instrumental support in both sexes and religion in men.

It is of interest that all sociodemographic variables showed significant negative correlations with stress in both sexes; this suggests that many other variables, mainly those related to constant conditions in life, including age, having a romantic partner, schooling, and having a job, also contribute to stress variability in both sexes. 

The study has the following limitations: the sample was not randomly selected and is mainly young, so the representativeness of the Mexican population is diminished and restricted to the young and people with more education. In addition, the low response rate (around 26%) also diminished the representativeness of the targeted population. On the other hand, the cross-sectional design of the study does not permit us to demonstrate causality, so the presence of bilateral relationships between the studied variables is also plausible; in this sense, it is possible that increased stressful conditions and or negative psychological variables (mainly in women) could also increase maladaptive coping strategies and diminish adaptive ones, as previously mentioned. In the case of women, it is possible that biological factors, including hormones and hormone response variations [7,8], contribute to a higher frequency of stress, depression, and anxiety, which, in turn, could increase maladaptive coping strategies and diminish adaptive ones, producing a positive refeeding cycle. However, with respect to the theoretical limitations, we consider that the results reported in this study are as expected, according to the classification of the strategies performed by Meyer, showing negative correlations between adaptive strategies and stress, depression, and anxiety as well as positive correlations between maladaptive coping strategies and stress, depression, and anxiety. Nevertheless, in this report, emotional strategies (emotional and instrumental support) seemed to be neutral (neither adaptive, nor maladaptive) according to the correlations found, and religion and humor showed different correlations with stress in each sex. 

In relation to objective number two, we also found a coincidence with the meta-analyses previously mentioned [28], which showed that the female sex was associated with the use of maladaptive coping strategies.

With reference to the implications for future research and potential intervention work, we consider that it is needed to perform experimental studies where subjects of both sexes are exposed to stressful situations in order to detect the coping strategies mainly used in each sex, along with the measurement of stress, depression, anxiety, and stress-coping strategies, before and after the experiment. These studies would be useful to corroborate the causal relationship between the variables analyzed in this study. Observational and longitudinal studies will also elucidate the causal relationship between stress-coping strategies and negative psychological variables in each sex. Finally, based on these results, it is important to mention that the implementation of intervention programs addressing an increase in emotional abilities, mainly emotional intelligence [29], that favor adaptive and diminish maladaptive coping, is needed and useful in order to diminish stress, depression, and anxiety. These programs should be administered at least from early education, as well as in labor and academic spaces with high-stress conditions. 

## 5. Conclusions

In conclusion, women showed higher frequencies of stress, depression, and anxiety than men and higher levels of some maladaptive coping (behavioral disengagement and denial) strategies and lower frequencies of some adaptive ones (active coping and planning) in comparison with men. This could explain the higher frequency of these negative psychological variables in women. The coping strategies showed similar correlations with stress, depression, and anxiety in both sexes with the exception of humor, which was shown to be adaptive in men and maladaptive in women, and religion, which seemed to be adaptive only in women. Therefore, based on these correlations, we could determine common and specific adaptive, maladaptive, and neutral coping strategies according to sex.

In addition, the most associated coping strategy with stress in both sexes was self-blame, which indicates that intervention programs addressed to diminish this strategy could be effective in diminishing stress, depression, and anxiety; however, the diminishing of maladaptive coping strategies (mainly avoidance coping strategies) and the increasing of adaptive ones, mainly active-coping, are also essential. Further experimental and longitudinal studies will determine the causal associations between stress coping strategies and stress, anxiety, and depression in both sexes but mainly in women. 

## Figures and Tables

**Table 1:** Sociodemographic variables in the studied population.

Variable	Men	Women	*p* Value
	N = 452	N = 831	
Age, mean ± SD	31.33 ± 11.60	31.47 ± 11.10	0.578
With romantic partner, n (%)	264 (58.40)	526 (63.30)	0.093
With job, n (%)	259 (57.30)	544 (65.50)	0.054
Educational level			
- Elementary school - High school - Preparatory - Bachelor’s degree - Technical career - Master’s degree - Ph.D. degree	1 (0.20) 7 (1.50) 96 (21.20) 249 (55.10) 22 (4.90) 46 (10.20) 31 (6.90)	1 (0.10) 14 (1.70) 156 (18.80) 459 (55.20) 38 (4.60) 119 (14.30) 44 (5.30)	0.328
Socioeconomic level			
- Very low - Low - Medium - High	4 (0.90) 68 (15.00) 365 (80.80) 15 (3.30)	0 (0.00) 130 (15.60) 677 (81.50) 24 (2.90)	0.07

SD: Standard deviation.

**Table 2:** Comparison of psychological variables and coping strategies between sexes.

Psychological Variable	Men (N = 452) Mean ± SD	Women (N = 831) Mean ± SD	*p* Value
**Stress**	2.68 ± 0.66	2.99 ± 0.64	<0.001
**Depression**	0.98 ± 0.74	1.30 ± 0.81	<0.001
**Anxiety**	0.99 ± 0.60	1.25 ± 0.65	<0.001
**Coping strategies (Brief-COPE)**			
**Self-blame**	1.28 ± 0.81	1.33 ± 0.84	0.415
**Behavioral disengagement**	0.46 ± 0.59	0.56 ± 0.61	0.001
**Self-distraction**	1.51 ± 0.81	1.58 ± 0.76	0.167
**Denial**	0.41 ± 0.62	0.53 ± 0.68	0.001
**Substance use**	0.30 ± 0.61	0.29 ± 0.64	0.232
**Emotional support**	1.11 ± 0.83	1.27 ± 0.82	<0.001
**Instrumental support**	1.21 ± 0.72	1.27 ± 0.73	0.317
**Active coping**	1.97 ± 0.70	1.88 ± 0.70	0.024
**Planning**	1.91 ± 0.76	1.75 ± 0.77	<0.001
**Acceptance**	1.99 ± 0.72	1.80 ± 0.68	<0.001
**Positive reframing**	1.56 ± 0.83	1.53 ± 0.75	0.586
**Religion**	0.81 ± 0.93	0.96 ± 0.91	<0.001
**Humor**	1.57 ± 0.94	1.31 ± 0.89	<0.001

The stress scale (CPSS) had a range of 1–5; the depression scale (CES-D) of 0–3; the anxiety scale (GAD-7) had a range of 0–3; and coping strategies (brief-COPE) had a range of 0–3.

**Table 3:** Correlation between coping strategies and stress, depression, and anxiety by sex.

Men (N = 452)				Women (N = 831)		
Coping Strategy	Stress	Depression	Anxiety	Stress	Depression	Anxiety
**Self-blame**	0.391 **	0.432 **	0.407 **	0.457 **	0.505 **	0.531 **
**Behavioral disengagement**	0.304 **	0.278 **	0.209 **	0.345 **	0.403 **	0.289 **
**Self-distraction**	0.238 **	0.302 **	0.264 **	0.143 **	0.222 **	0.217 **
**Denial**	0.227 **	0.235 **	0.170 **	0.328 **	0.402 **	0.336 **
**Substance use**	0.216 **	0.242 **	0.203 **	0.186 **	0.248 **	0.213 **
**Emotional support**	0.067	0.053	0.047	−0.023	0.006	0.061
**Instrumental support**	−0.002	0.003	0.052	0.048	0.030	0.114 **
**Active coping**	−0.327 **	−0.261 **	−0.146 **	−0.276 **	−0.210 **	−0.069 **
**Planning**	−0.318 **	−0.248 **	−0.140 **	−0.268 **	−0.219 **	−0.090 **
**Acceptance**	−0.210 **	−0.166 **	−0.133 **	−0.257 **	−0.217 **	−0.150 **
**Positive reframing**	−0.141 **	−0.148 **	−0.071	−0.217 **	−0.157 **	−0.092 **
**Religion**	−0.027	−0.026	0.040	−0.204 **	−0.209 **	−0.156 **
**Humor**	−0.097 *	−0.048	−0.015	0.081 *	0.111 **	0.120 **

* *p* < 0.05, ** *p* < 0.01. *p* value obtained with the Spearman correlation test.

**Table 4:** Bivariate correlations between sociodemographic variables and stress.

Variable	Men (N = 452)	Women (N = 831)
**Age**	−0.174 **	−0.302 **
**Schooling**	−0.141 **	−0.150 **
**Having children**	−0.198 **	−0.199 **
**With romantic partner**	−0.147 **	−0.163 **
**With job**	−0.100 *	−0.202 **
**Socioeconomic level**	−0.182 **	−0.114 **

* *p* < 0.05, ** *p* < 0.01. *p* value obtained with the Spearman correlation test.

**Table 5:** Multiple regression analysis for stress in women.

Variable	B	Beta Coefficient	*p* Value	Change in R^2^
**Constant**	3.387	-	<0.001	-
**Self-blame**	0.256	0.335	<0.001	0.214
**Active coping**	−0.139	−0.152	<0.001	0.078
**Age**	−0.008	−0.136	<0.001	0.036
**Denial**	0.106	0.111	<0.001	0.024
**Acceptance**	−0.146	−0.154	<0.001	0.020
**Have a job**	−0.119	−0.088	0.003	0.009
**Self-distraction**	0.086	0.102	0.001	0.006
**Positive reframing**	−0.089	−0.104	0.002	0.007
**Behavioral disengagement**	0.092	0.088	0.005	0.006

Type of method for the regression model: Stepwise. R of the model = 0.633.

**Table 6:** Multiple regression analysis for stress in men.

Variable	B	Beta Coefficient	*p* Value	Change in R^2^
**Constant**	3.387	-	<0.001	-
**Self-blame**	0.296	0.367	<0.001	0.176
**Active coping**	−0.184	−0.196	<0.001	0.114
**Denial**	0.109	0.104	0.009	0.024
**Acceptance**	−0.093	−0.102	0.024	0.019
**Self-distraction**	0.158	0.196	<0.001	0.030
**Humor**	−0.109	−0.157	<0.001	0.015
**Substance use**	0.133	0.125	0.001	0.014
**Socioeconomic level**	−0.172	−0.118	0.002	0.008
**Schooling**	−0.085	−0.099	0.045	0.005

Type of method for the regression model: Stepwise. R of de model = 0.637.

## Data Availability

Data that support the findings of the study are available upon a reasonable request.
